# Making connections: the scientific impact and mentoring legacy of Dr. John E. Moulder

**DOI:** 10.1080/09553002.2023.2176563

**Published:** 2023-02-15

**Authors:** Andrea L. DiCarlo, David R. Cassatt, Carmen I. Rios, Merriline M. Satyamitra, Yuji Zhang, Trevor G. Golden, Lanyn P. Taliaferro

**Affiliations:** a Radiation and Nuclear Countermeasures Program; Division of Allergy, Immunology, and Transplantation; National Institute of Allergy and Infectious Diseases, National Institutes of Health, Rockville, MD, USA; b Department of Epidemiology and Public Health, Marlene and Stewart Greenbaum Comprehensive Cancer Center, University of Maryland School of Medicine, Baltimore, MD, USA

**Keywords:** Education, training, government, NIH, funding, radiation, biology, legacy

## Abstract

**Purpose:**

The intent of this mini review is to pay homage to Dr. John E. Moulder’s long and successful career in radiation science with the Medical College of Wisconsin. This effort will be done from the perspective of his history of U.S. Government funding for research into the biological pathways involved in radiation-induced normal tissue injuries, especially damage to the kidneys and heart, and pharmacological interventions. In addition, the impact of his steady guidance and leadership in the mentoring of junior scientists, and the development of meaningful collaborations with other researchers will be highlighted.

**Conclusion:**

Dr. John E. Moulder’s contributions to the field of radiation research, through his strong character and reputation, his consistent and dedicated commitment to his colleagues and students, and his significant scientific advances, have been critical to moving the science forward, and will not be forgotten by those who knew him personally or through publications documenting his important work.

## Introduction

**Figure 1.**
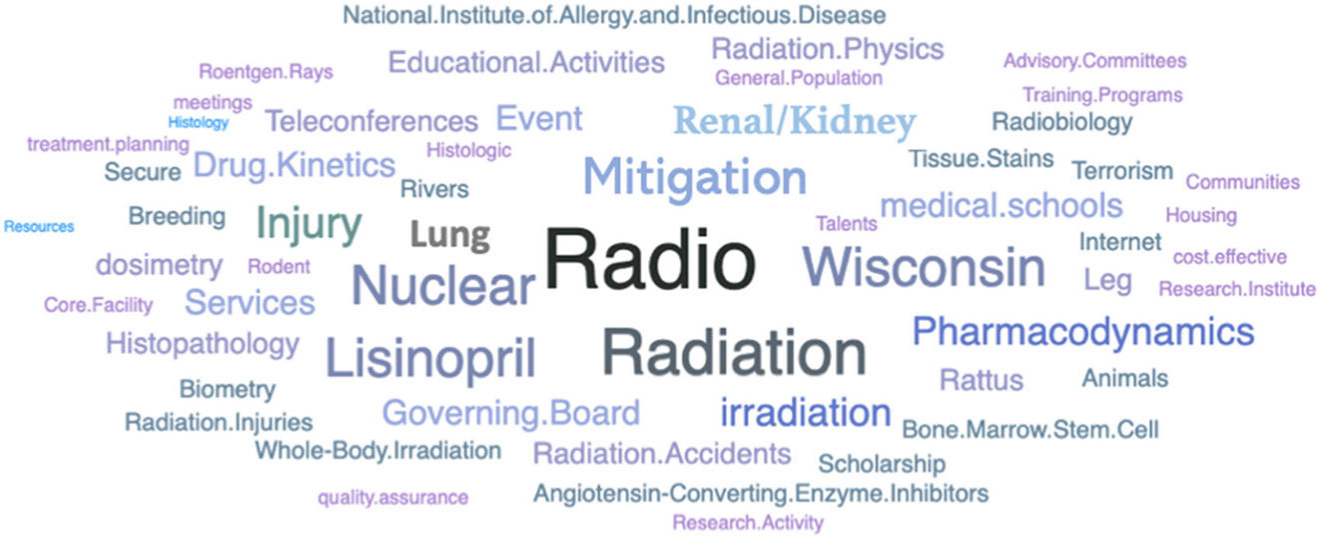
Research, Condition, and Disease Categorization (RCDC) Word Cloud for John E. Moulder’s NIH grants portfolio. The RCDC is a computer-based process that compiles a list of all the NIH-funded grants and contracts into specific categories such as research area, disease, or condition.

**Figure 2.**
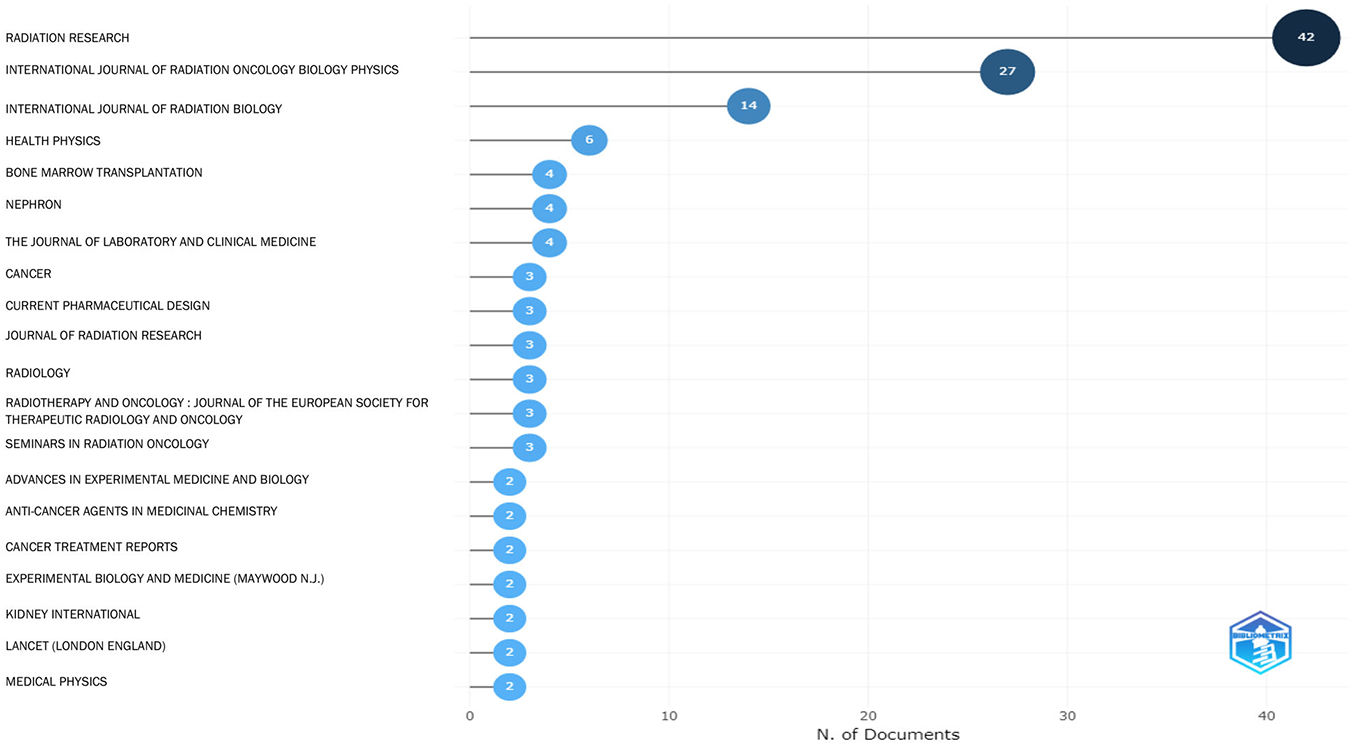
John Moulder’s list of publications sorted by journal names. The number in the oval at the end of each line segment represents the number of publications in the indicated scientific journal. The size and darkness of the oval are proportional to the publication number.

Although it is difficult to encompass all of the scientific contributions of Dr. John E. Moulder (1945–2022), this paper intends to look at the research funding that Dr. Moulder brought to the Medical College of Wisconsin (MCW) during his 35-year career. This manuscript will focus on the impact that this funding and John’s many mentorships and collaborations had on his colleagues and the next generation of investigators. Dr. Moulder was an active researcher from 1969 to 2021, receiving various National Institutes of Health (NIH) grant and cooperative agreement awards (e.g. R01 and U19; see Figure 1 for high-level topic overview) along with non-NIH awards, which resulted in 188 peer-reviewed publications with over 150 individuals in a variety of well-respected journals. Dr. Moulder coauthored a quarter of these publications as the principal investigator for a U19 grant under the National Institute of Allergy and Infectious Diseases (NIAID) Centers for Medical Countermeasures against Radiation (CMCR). The top three journals for his work included Radiation Research (where John also served as a Senior Editor), International Journal of Radiation Oncology, Biology and Physics, and International Journal of Radiation Biology (Figure 2). He also received considerable NIH funding from the National Cancer Institute (NCI), where he held a long-term project focused on the effects of cancer chemo- and radiotherapy on the kidney. His funding; however, was not limited to the NIH. For example, Dr. Moulder collaborated with MCW colleague John Baker in 2014 on a multi-year award from NASA to study the risk of heart disease from space radiation exposure.

John’s funding also included private foundations. In 2013, he received support from the Wi-Fi Alliance to study risks that might be attributable to WiFi signal exposure (Foster and Moulder 2013, 2015). In similar work, he played an important role in better understanding other forms of radiation, namely non-ionizing exposures. This included radiofrequency and extremely-low-frequency electromagnetic fields (EMF), such as those emitted by mobile telephones (Moulder et al. 1999; Khurana et al. 2008), cell phone towers (Moulder et al. 2005), power lines (Moulder and Foster 1995; Moulder 1997, 1998; Moulder and Foster 1999; Moulder 2000), or airport scanners (Moulder 2012). He showcased the latter expertise in a Radiation Research Society (RRS) videocast titled ‘Risk of Exposure to Ionizing and Millimeter Wave Radiation from Airport Scanners’. John also took an early stance on hormesis in 1998 (Cameron and Moulder 1998), and later published on the risks from medical imaging using radiation, (Foster et al. 2017) and chemical radio-sensitizers (Moulder 2019). During the early 1990s, John served on panels to establish radiation protection standards (Moulder 1992), and his participation in an inter-agency workshop in Bethesda in 2001, organized by Drs. C. Norman ‘Norm’ Coleman and Helen Stone (Stone et al. 2002), led the way for the development of what would later become the NIAID Radiation and Nuclear Countermeasures Program (RNCP) (Moulder 2002a, 2002b), from which many of these authors hail. Once he became an NIAID RNCP awardee, John continued his interest in radiation public health emergencies, participating in the 2009 Red Dragon Drill – a tabletop exercise for the detonation of a nuclear device in Milwaukee (Martz et al. 2011). In fact, John’s subject matter expertise was further recognized by the U.S. Government through their acknowledgment of his consulting reviewer role on the Department of Health and Human Services’ Radiation Emergency Medical Management (REMM) website, which provides online guidance to health care personnel on diagnosis and treatment of radiation injuries^1^.

John had the tenacity and vision to persist in solving the clinical problem of radiation-induced lung toxicity, eagerly sharing ideas with colleagues and thereby demonstrating to young investigators how collaborative science is truly done.

– C. Norman Coleman

## A history of NIH research excellence – the National Cancer Institute years

For 22 consecutive years, Dr. Moulder held an R01 research grant awarded by the NCI within the NIH titled *Kidney Response to Radiation and Chemotherapy.* The project, established in 1979, was conducted in the MCW Department of Radiation Oncology to explore if late radiation-induced normal tissue injuries could be prevented and/or treated with post-irradiation pharmacologic intervention. The overarching goal of Dr. Moulder’s R01 project was to use rodent models to study biological mechanisms involved in late radiation-induced tissue injuries. The proposal was based on the discovery that radiation nephropathy is a major complication of total body irradiation (TBI), a procedure used in bone marrow transplantation (BMT). The Moulder laboratory successfully developed an irradiated rat model that accurately simulates the nephropathy noted in human patients. In addition, the discovery that angiotensin converting enzyme (ACE) inhibitors and angiotensin II (AII) blockers could be used as a treatment for BMT nephropathy laid the groundwork for these critical studies. As such, the project was strongly influenced by the finding that ACE inhibitors and AII blockers could permanently interfere with the development of BMT-associated nephropathy, even when treatment was started after irradiation.

John’s findings challenged what was known at the time regarding mechanisms of late radiation injuries and opened the possibility that normal tissue radiation injuries could be treated, or even prevented, by post-irradiation interventions. To address this hypothesis, Dr. Moulder focused on the following specific aims throughout the course of the 20+ year R01 project: 1) demonstrate radiation-induced activation of the renin-angiotensin system (RAS) as the cause of radiation- (and BMT-) induced nephropathy; 2) show that fibrosis was a cause, rather than a consequence, of radiation nephropathy; 3) confirm that angiotensin 1 (AT1) receptor antagonists were preferentially effective in the prophylaxis and treatment of radiation (and BMT) nephropathy; 4) retrospectively assess factors influencing incidence of BMT nephropathy in humans; 5) determine in a prospective randomized clinical trial whether captopril could be used to lower the incidence of BMT nephropathy; 6) assess the basis for dependence of renal tolerance on age-at-irradiation, and whether the change in tolerance was related to the decreased effectiveness of ACE inhibitors in older animals; and 7) identify conditions under which pulsed and continuous low dose rate irradiation produce clinically equivalent normal tissue injuries.

From this single R01 project, 64 publications have been generated to date. John shared authorship with over 80 distinct collaborators from institutions across the United States, in journals that spanned the fields of cancer and radiobiology, including the Lancet, the International Journal of Radiation Oncology, Biology, Physics, and Radiation Research (among many others – See Figure 2). The lion’s share of his authorships included Eric Cohen (NYU Langone University Medical Center) who appeared on 48 of them.

John was a friendly mentor and senior colleague who provided critiques and support that I value to this day.

– Eric P. Cohen

In addition, preclinical work conducted through this R01 would later inform an associated clinical trial led by Dr. Cohen to study mechanisms of late radiation-induced tissue injury in humans. The study enrolled 55 subjects from 1998 to 2006: including 52 adults and 3 children. Eligible candidates were patients over the age of six years receiving autologous marrow or peripheral stem cells from siblings or unrelated donors. Only patients receiving myeloablative TBI as part of the preparative regimen were included. Data generated through this study would extend an initial report of consistent trends for benefit of captopril to mitigate radiation injury after TBI-based hematopoietic stem cell transplant. The study identified the occurrence of the BMT nephropathy syndrome as being less in subjects who received captopril compared to placebo (Cohen et al. 2012).

Dr. Moulder also examined radiosensitizers in a grant funded by the NCI in 1980. This grant, along with grants to his collaborators, spurred a successful partnership with Drs. Sara Rockwell and Douglas Martin of Yale University. An important factor of tumor response to radiotherapy is the degree of hypoxia present in the tumor; hypoxic areas of a solid tumor tend to be more radioresistant [reviewed by Moulder and Rockwell (1987)], and understanding the pathways behind hypoxia induction of tumor resistance is helping lead to the development of radio-sensitizing agents.

In a series of studies, John and collaborators sought to quantify hypoxia and the relationship between the extent of hypoxia and tumor resistance. They first needed to establish and quantify the degree of hypoxia in tumors. In their most-cited paper supported by this NCI grant, they reviewed the literature describing hypoxia in various tumor types and sizes in rodents (Moulder and Rockwell 1984) and found that as tumors grew from microscopic to macroscopic, the area of hypoxia increased, although tumor growth beyond the macroscopic stage did not appear to result in a greater hypoxic fraction. Furthermore, the literature review revealed that radiation response in rodents did not appear to correlate with hypoxic fraction (Rockwell et al. 1984). In this grant, the teams also examined intratracheal tumor implantation in rats (Byhardt et al. 1984), hypoxia measurement techniques (Moulder and Martin 1984), and methods of hypoxia induction (Rockwell et al. 1986). Beyond the slower division of cells in hypoxic regions, the mechanisms of hypoxia-associated tumor resistance have since been shown to involve hypoxia-inducible factors [reviewed Jing et al. (2019)], and these factors have been proposed as targets to sensitize tumors to radiotherapy (Wigerup et al. 2016).

## The Center for Radiation Injury Intervention (CENTRII) at Medical College of Wisconsin – NIAID-funded CMCR

**Figure 3.**
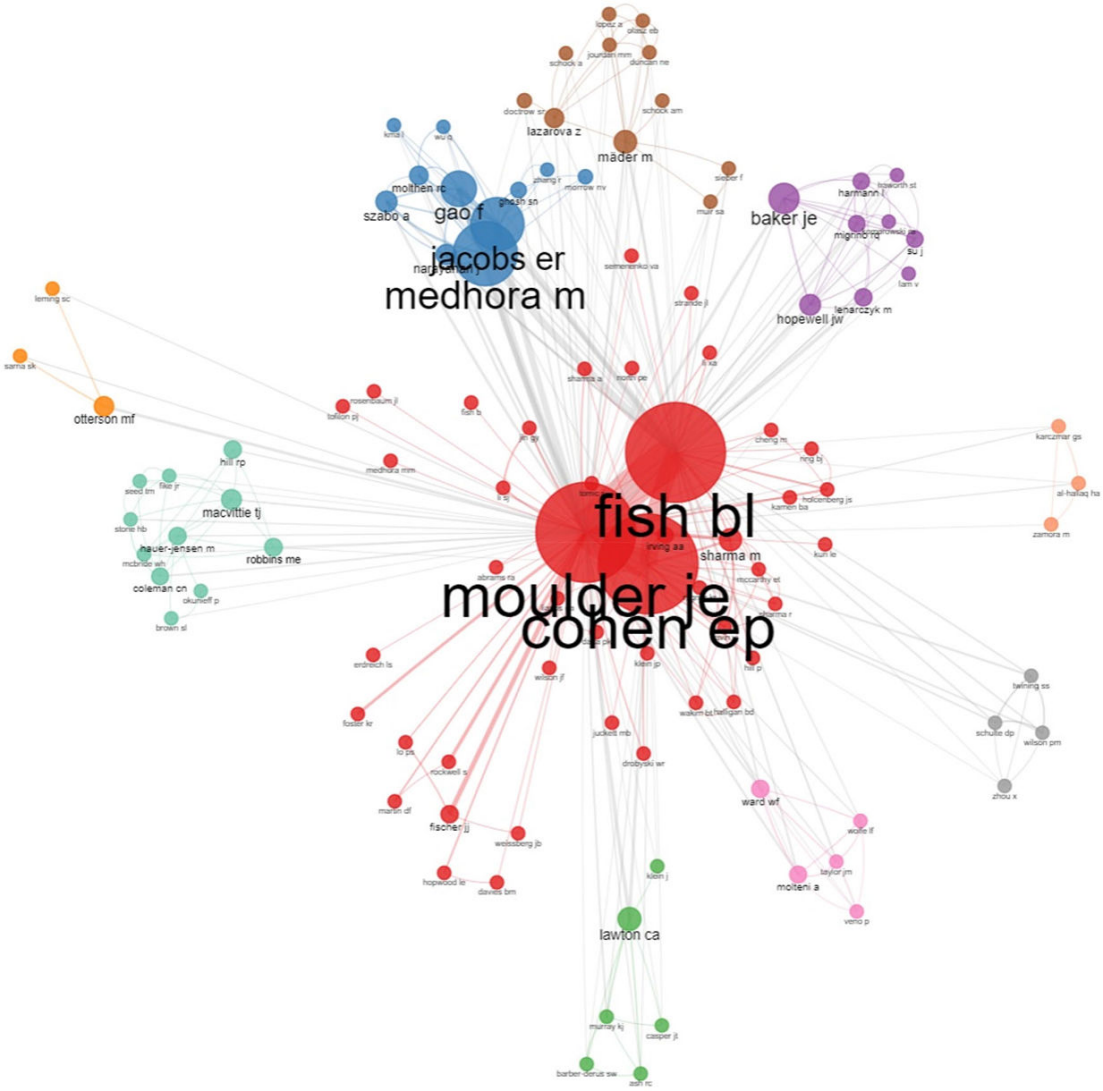
John Moulder’s Collaborative Publication Network 1969–2021. 188 publications; >150 authors; 69 journals. The R package Bibliometrix was used to generate the collaboration network, using default parameters (Massimo and Cuccurullo 2017).

Established in 2005, CENTRII at MCW was awarded $3.7 million by the NIAID in its first year of funding, as one of eight Centers of Excellence for Radiation Research (RFA-AI-04-045)^2^. The CMCR program is a network of national research centers working together to develop effective and comprehensive medical countermeasures applicable to all subsets of the civilian population in the event of radiological or nuclear emergencies. They utilize multidisciplinary basic and translational research to support the development of new medical products that will assess, diagnose, mitigate and/or treat the short- and long-term consequences of radiation exposure after a radiological/nuclear terrorist event or accidental exposure. Dr. Moulder had a robust collaboration network including CMCR investigators and others outside of the CMCR (Figure 3). Within CENTRII, he led a team of investigators including Drs. Meetha Medhora, Eric Cohen, Makut Sharma, Parvenah Rafiee, and Brian Fish in a project to study *Post-Irradiation Intervention to Mitigate and Treat Chronic Renal Injuries* (red cluster. Figure 3). Within this team, Dr. Moulder published extensively with Brian Fish, with over 86 publications together.

John inspired, taught, and mentored me. He changed my life and changed the world’s thinking on radiation injuries. His many contributions to the field will never be forgotten

John, my friend, will be missed.

– Brian Fish

John’s CMCR network also extended to Drs. Meetha Medhora, Elizabeth Jacobs, Timothy Lowry, and Robert Molthen, who led the study on *Modulation of Post-Irradiation Changes in Pulmonary Vasculature* (Figure 3, blue cluster). Another hub on his CMCR network included Dr. Mary Otterson who focused on the *Post-Irradiation Modulation of Gastrointestinal Injury* (Figure 3, orange cluster). Other non-CMCR areas of interest included in Figure 3 include radiation cardiac injury (purple cluster), radiation effects on immune function (grey cluster), cutaneous radiation injury (brown cluster), radiation animal models (turquoise cluster), radiation renal injuries (pink and green clusters), and tumor carcinogenesis (peach cluster).

Notable post-docs within the CMCR publication network include: 1) Dr. Sreedhar Bodiga, who studied the modulation of post-irradiation changes in pulmonary vasculature under Dr. Meetha Medhora. In 2022, he was an Associate Biochemistry Professor at Forest College & Research Institute in India studying intestinal physiology, oxidative stress disorders, and cardiovascular biology, and 2) Dr. Javed Mahmood, who worked under Dr. Patrick Hill in the MCW Center, studying radiation effects of lung tissue. As of late 2022, Javed serves as a Clinical Study Director and Senior Clinical Scientist at Bristol Myers Squibb Pharmaceutical working on lung cancer clinical trials.

Having a scientist of John’s stature as a mentor and a friend, greatly boosted my confidence and gave me a sense of security.

– Meetha Medhora

## Mentoring the next generations: training and education efforts

Although formalized with his administrative oversight of the MCW U19 Training and Education Core, John’s impact on training the next generation of radiation biologists was far-reaching by the time he was awarded a NIAID Center in 2005. In that funding, John, alongside many of the Center’s senior scientists (including researchers from Henry Ford, University of Toronto, and Eukarion, Inc.), focused on post-doctoral trainees who were a part of projects within the Center and other MCW faculty with primary training in areas outside of radiation. His Center offered both hands-on laboratory training in assays, methods, reagents, models, technologies, etc., and a didactic program involving seminars on radiation-induced normal tissue responses and injuries from experts in the field.

The lecture series housed on the MCW Center website (www.centrii.org; now inactive) was provided as real-time training online and as videos for later viewing and was accompanied by self-test modules to ensure comprehension of the material. In a move that was obviously ahead of its time and that foreshadowed what research might look like over a decade later, the first webcast was posted in 2006. These valuable videos allowed students and researchers within other CMCR awardee sites to access both basic as well as cutting-edge radiation science. Trainees were also exposed to journal clubs, institutional speakers, and ongoing lectures provided for radiation oncology medical students, to help them understand the context of medical countermeasures and model development. During the U19 award period, seven trainees benefited from resources provided by the program and were able to parlay their experiences into presentations at meetings of the RRS, the International Congress of Radiation Research, and the American Society for Therapeutic Radiation Oncology. Most of those fellows that were supported by the MCW Core continue to publish papers in the radiation field (Prevost et al. 2018; Lenarczyk et al. 2019; Wunderle et al. 2019; Lenarczyk et al. 2020; Leduc et al. 2021; Raber et al. 2021; Hsu et al. 2022; Liu et al. 2022) and obtain follow-on funding (e.g. pilots or full research grants). In addition, several went on to establish their own research labs, were promoted to full faculty, or went into private practice.

## Expanding the radiation research field through pilot project awards

In addition to his stellar research work in oncology and radiation biology, Dr. Moulder’s significant role in recruiting new scientists into the radiation research field went beyond his training programs. New investigators were also brought in through the Pilot Grants Program under the MCW CMCR U19 grant. The goals of the MCW Pilot Grant Program were to:

1. Encourage exploratory and developmental research projects relevant to the MCW CMCR mission by providing support for research projects during their early and conceptual stages of development;
2. Facilitate development and incorporation of new technologies into the MCW CMCR; and
3. Foster collaboration between members of the different MCW CMCR programs.

Pilots were awarded in years two through five (renewals only in the final year) of the CMCR timeline. From the beginning, this application attracted a large number of high-quality applications. Throughout the MCW Center award period, 49 applications/renewals were considered, and 29 one- or two-year awards were made.

Perhaps the most important feature of these pilot awards was that more than half of the awardees were not directly involved in radiation research before receiving pilot funds. These awards introduced excellent investigators from other research areas to radiation science and enhanced their contributions to the field. Bringing diverse skill sets and fresh ideas that sparked new research avenues for radiation biology, the pilot PIs represented some of the best and the brightest. Their contributions toward advancing novel research are evidenced by publications in top journals and presentations at widely attended national and international conferences. Further, most of the pilot awardees covered a wide range of CMCR topics and brought expertise not only from radiation oncology and radiology groups, but also from a wide range of other specialties including pediatric surgery, gastroenterology, nephrology, and pathology.

Highlights from the awarded pilot projects included: 1) finding that cardiac injury occurs in a rat model after TBI, but not after whole thorax irradiation (Lenarczyk et al. 2013); and confirmation that this injury can be mitigated by simvastatin (Lenarczyk et al. 2015); 2) development of a new series of orally bioavailable SOD/catalase mimetics (Vorotnikova et al. 2010); 3) evidence that TBI and local renal irradiation cause changes in the urine proteome within 24 hours (Sharma et al. 2010); 4) development of software to allow a clinical SPECT/CT system to map total-body dose from internally deposited isotopes of concern for radiological terrorism (Medhora et al. 2016); 5) demonstration that dietary selenium supplementation can provide a simple, safe, and effective way of mitigating renal injury induced by a single TBI dose of 10 Gy (Sieber et al. 2011); and 6) establishment that radiation-induced skin and lung injury can be mitigated by manganese superoxide dismutase gene therapy (Yan et al. 2008). At least two of the pilot research projects were also identified as sufficiently promising by the MCW CMCR Coordinating Committee as to be considered for elevation to the level of a full project within the Center. The data generated in a few of these pilots also resulted in new R01 and U01 awards.

## Conclusion

Through his awarded research grant and cooperative agreements from the NIH and beyond, John leaves behind a legacy of excellent, rigorous, and robust scientific findings, research collaborators who benefited from his expertise and dedication, and a cadre of well-trained students. Although it is impossible to list here all the lives that were touched, and the careers that were impacted by John’s influence, the authors can state with certainty that the field of medical preparedness for a radiation public health emergency would not be where it is now without the steadying hand and role played by Dr. Moulder, both in the early days in the program and during his final years as an active researcher. We are grateful for his years of research and join the entire radiation community in mourning the loss of a great investigator and person.

## Footnotes

1.
https://remm.hhs.gov/Aboutthissite.htm

2.
https://grants.nih.gov/grants/guide/rfa-files/RFA-AI-04-045.html
